# Supplementary material for: The Deep Proteomics Approach Identified Extracellular Vesicular Proteins Correlated to Extracellular Matrix in Type One and Two Endometrial Cancer
Source: Int J Mol Sci. 2024 Apr 24;25(9):4650. doi: 10.3390/ijms25094650 (PMC11083465; doi:10.3390/ijms25094650)
Supplement: Supplementary file 1 [file ijms-25-04650-s001.zip › Figure S1.pdf]

Whole membrane stained with red ponceau

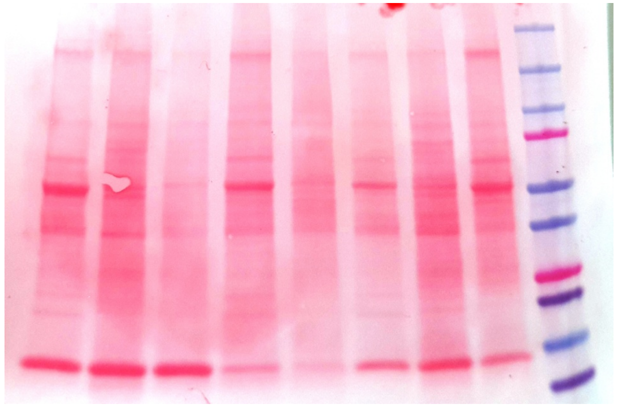

Tissue lysate membrane 1

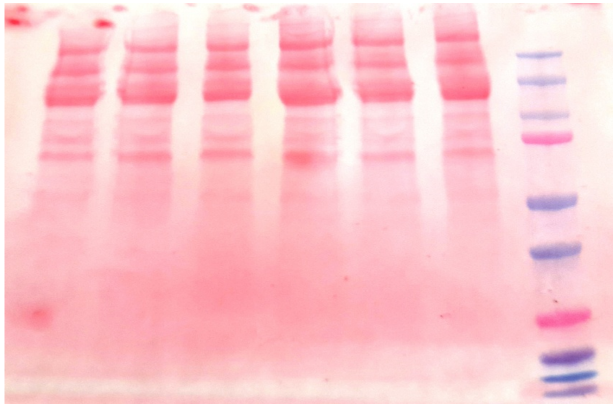

Cell lysate membrane

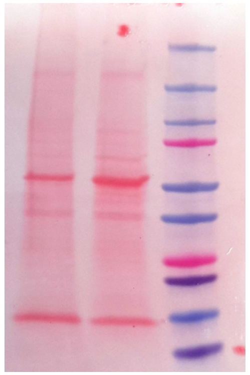

Tissue lysate membrane 4

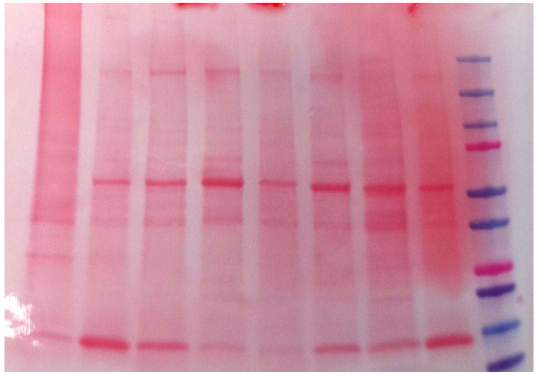

Tissue lysate membrane 3

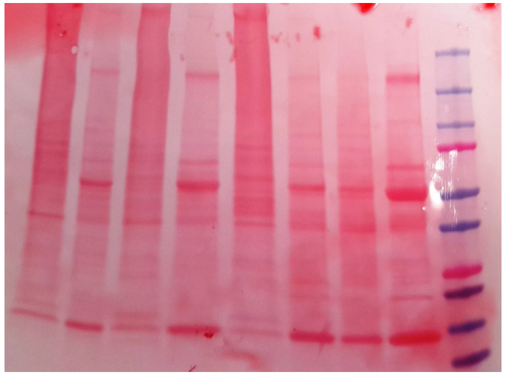

Tissue lysate membrane 2

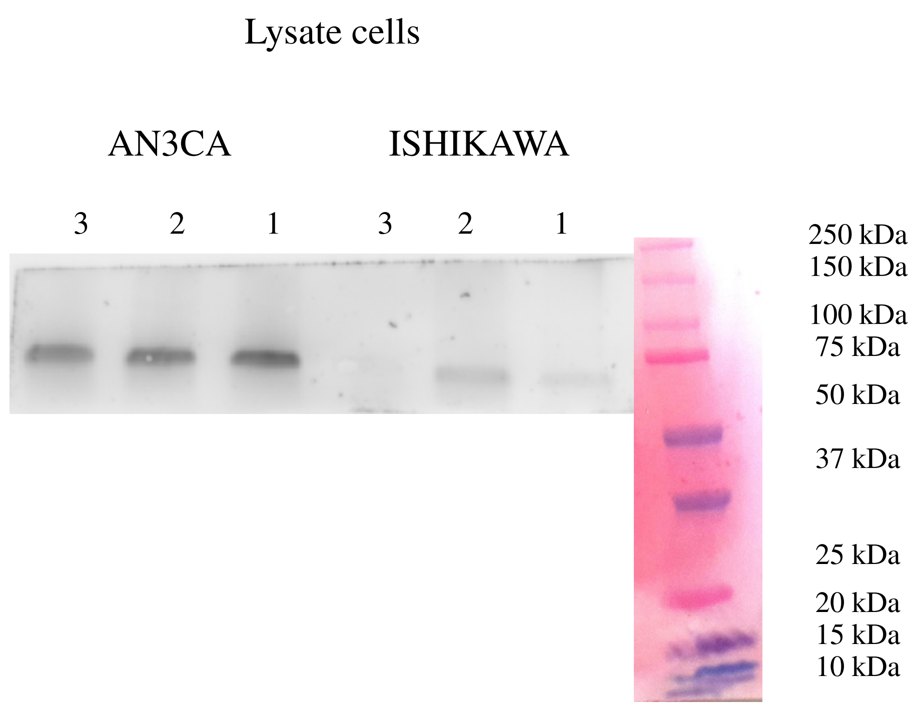
Lysate cells
AN3CA
ISHIKAWA
3
2
1
3
2
1
250 kDa
150 kDa
100 kDa
75 kDa
50 kDa
37 kDa
25 kDa
20 kDa
15 kDa
10 kDa

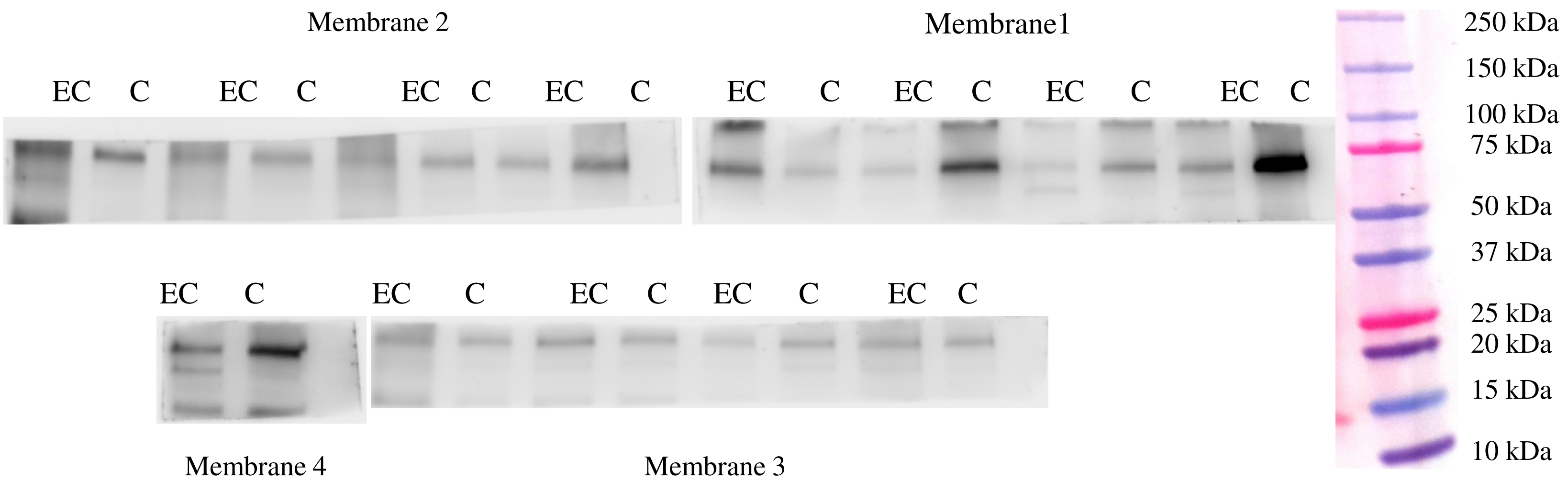

Whole membrane of MMP2 protein

C-control
EC- endometrial cancer

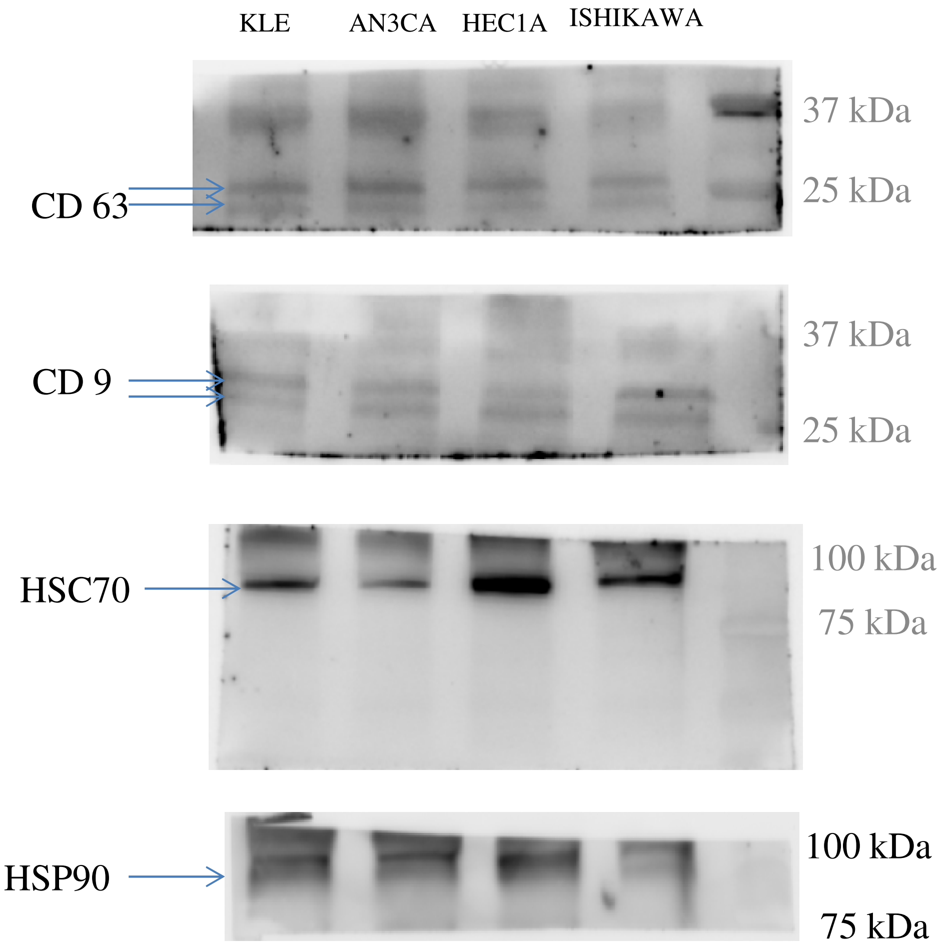
KLE
AN3CA
HEC1A
ISHIKAWA
CD 63
37 kDa
25 kDa
CD 9
37 kDa
25 kDa
HSC70
100 kDa
75 kDa
HSP90
100 kDa
75 kDa
