# Supplementary material for: The Deep Proteomics Approach Identified Extracellular Vesicular Proteins Correlated to Extracellular Matrix in Type One and Two Endometrial Cancer
Source: Int J Mol Sci. 2024 Apr 24;25(9):4650. doi: 10.3390/ijms25094650 (PMC11083465; doi:10.3390/ijms25094650)
Supplement: Supplementary file 1 [file ijms-25-04650-s001.zip › Table S6.pdf]

Supplementary Table S6: Clinico-pathological characteristics of the 13 EC women enrolled in the study.

Sample number	Age	Histotype (Grade)	Tumor dimensions (cm)	TNM Classification of Malignant Tumours, 7 Edition	Stage	ICD-11
80	70	Endometroid adenocarcinoma (G2)	2x1,7	pT1b N0 G2 LVI+	I B	2C76
177	79	Endometroid adenocarcinoma (G1)	4.8x3.8	pT1b G1 LVI+ R0	IB	2C76
45	75	Endometroid adenocarcinoma (G2)	3	pT1b G2 Nx	I B	2C76
190	56	Endometroid adenocarcinoma (G1)	3 x 2.5 x 0.5	pT1a N0(sn)(i-) G1 LVI- R0	IA	2C76
168	72	Endometroid adenocarcinoma (G3)	Macroscopically not evaluatable	pT1a N0 G3 LVI - R0	IA	2C76
130	52	Endometroid adenocarcinoma (G1)	4.6	pT1a G1 LV- R0	IA	2C76
124	72	Endometroid adenocarcinoma (G2)	2.5	pT1b N0 G2 LVI- R0	I B	2C76
185	60	Serous carcinoma (G3)	2x0.7	pT1a N0 G3 V0 Pn0 R0	IA	2C76
238	83	Endometroid adenocarcinoma (G3)	2.7x2x2.4	pT1b N1 M1 LVI +	IV A	2C76
179	81	Endometroid adenocarcinoma (G2)	5.2x4.2x1.3	pT2 Nx V1 Pn0 R0	II	2C76
119	87	Endometroid adenocarcinoma + papillary serous carcinoma (G3)	3x3	pT3a G3 LVI+ R1.	II	2C76
63	73	Papillary serous carcinoma (G3)	4x3x3	pT1b N0 G3 LVI+	I B	2C76
169	56	Endometroid adenocarcinoma (G3)	5	pT3b N1 G3 LV1 R1	IIIC1	2C76
