# Supplementary material for: The Deep Proteomics Approach Identified Extracellular Vesicular Proteins Correlated to Extracellular Matrix in Type One and Two Endometrial Cancer
Source: Int J Mol Sci. 2024 Apr 24;25(9):4650. doi: 10.3390/ijms25094650 (PMC11083465; doi:10.3390/ijms25094650)
Supplement: Supplementary file 1 [file ijms-25-04650-s001.zip › Table S7.pdf]

Supplement Table S7. Clinico-pathological characteristics of the 13 controls women enrolled in the study

**Sample type**	**Age**	**Diagnosis**	**ICD-11**
Control	47	Leiomyoma of uterus	2E86.0
Control	42	Leiomyoma of uterus	2E86.0
Control	45	Leiomyoma of uterus	2E86.0
Control	40	Leiomyoma of uterus	2E86.0
Control	32	Leiomyoma of uterus	2E86.0
Control	44	Leiomyoma of uterus	2E86.0
Control	47	Leiomyoma of uterus	2E86.0
Control	42	Leiomyoma of uterus	2E86.0
Control	47	Leiomyoma of uterus	2E86.0
Control	41	Leiomyoma of uterus	2E86.0
Control	51	Leiomyoma of uterus	2E86.0
Control	42	Leiomyoma of uterus	2E86.0
Control	45	Leiomyoma of uterus	2E86.0
